# The Dental Student’s Note Book

**Published:** 1880-03

**Authors:** 


### THE DENTAL STUDENT'S NOTE BOOK.

Is a little volume of 130 pages, containing within its flexible covers a large and varied amount of information pertaining to the development, structure and comparative anatomy of the teeth. The bulk of the work is devoted to pathology and surgery, and very properly.

The book is intended for ready reference by the student, both to refresh his memory and fix in his mind the material gathered in the lecture and clinic rooms. It is neat and attractive in form and style, being printed upon good paper. The chief objection to note books and vest-pocket encyclopedias in general, is the tendency they frequently exert to make the student shirk the solid work of the text books, and the seductive temptation to "pony" at the final examination.

PRESLEY BLAKISTON, Publisher.

To be had of Robt. Clarke & Co.

